# Effectiveness and cost-effectiveness of telehealthcare for chronic obstructive pulmonary disease: study protocol for a cluster randomized controlled trial

**DOI:** 10.1186/1745-6215-15-178

**Published:** 2014-05-21

**Authors:** Flemming Witt Udsen, Pernille Heyckendorff Lilholt, Ole Hejlesen, Lars Holger Ehlers

**Affiliations:** 1 Danish Centre for Healthcare Improvements, Aalborg University, Fibigerstraede 11, 9220 Aalborg Ø, Denmark; 2 Department of Health Science and Technology, Aalborg University, Fredrik Bajers Vej 7, C1-211, 9220 Aalborg Ø, Denmark

**Keywords:** Chronic obstructive pulmonary disease, Telemedicine, Cluster randomized trial, Effectiveness, Cost-effectiveness, Denmark

## Abstract

**Background:**

Several feasibility studies show promising results of telehealthcare on health outcomes and health-related quality of life for patients suffering from chronic obstructive pulmonary disease, and some of these studies show that telehealthcare may even lower healthcare costs. However, the only large-scale trial we have so far - the Whole System Demonstrator Project in England - has raised doubts about these results since it conclude that telehealthcare as a supplement to usual care is not likely to be cost-effective compared with usual care alone.

**Methods/Design:**

The present study is known as ‘TeleCare North’ in Denmark. It seeks to address these doubts by implementing a large-scale, pragmatic, cluster-randomized trial with nested economic evaluation. The purpose of the study is to assess the effectiveness and the cost-effectiveness of a telehealth solution for patients suffering from chronic obstructive pulmonary disease compared to usual practice. General practitioners will be responsible for recruiting eligible participants (1,200 participants are expected) for the trial in the geographical area of the North Denmark Region. Twenty-six municipality districts in the region define the randomization clusters. The primary outcomes are changes in health-related quality of life, and the incremental cost-effectiveness ratio measured from baseline to follow-up at 12 months. Secondary outcomes are changes in mortality and physiological indicators (diastolic and systolic blood pressure, pulse, oxygen saturation, and weight).

**Discussion:**

There has been a call for large-scale clinical trials with rigorous cost-effectiveness assessments in telehealthcare research. This study is meant to improve the international evidence base for the effectiveness and cost-effectiveness of telehealthcare to patients suffering from chronic obstructive pulmonary disease by implementing a large-scale pragmatic cluster-randomized clinical trial.

**Trial registration:**

Clinicaltrials.gov, http://NCT01984840, November 14, 2013.

## Background

The World Health Organization (WHO) has estimated that globally more than 64 million people have chronic obstructive pulmonary disease (COPD) and that more than 3 million people died of COPD in 2005, which was 5% of total deaths that year [1]. The prevalence of COPD in Denmark is among the highest in the world [2]. It is estimated that COPD affects 430,000 Danish citizens, that is, 7% of the entire population and 14% of citizens older than 35 years [3]. It is also estimated that around 5,000 citizens die due to COPD each year, which makes COPD the fourth largest cause of death in the country [4].

COPD is one of eight diseases that have been given priority by the Danish Health and Medicines Authority since 2002. Around 10 percent of the total annual Danish healthcare budget for citizens over 45 years is related to care and treatment of COPD; the costs are in excess of £300 million per year [4].

Recent systematic reviews show promising results of telehealthcare on health outcomes and health-related quality of life in COPD patients [5-7], and some feasibility studies indicate that the use of telehealthcare may lower the costs of such care [8-13]. These studies have fuelled clinical and political interest in telehealthcare as a way of improving COPD patient outcomes while cutting costs. However, there has been a call for large-scale clinical trials with rigorous cost-effectiveness assessments in telehealthcare research [14], and concerns about the quality of the current evidence have been raised [7,14]. The studies have a limited number of participants (typically fewer than 200) and have not been conducted in routine clinical practice. In addition, the evidence base on costs could be improved, and the studies usually do not draw conclusions on the cost-effectiveness of telehealthcare. This hampers a comparison of the expected health benefits and costs from telehealthcare with those that may be obtained from the use of other health technologies. Finally, in the recently published ‘Whole System Demonstrator Project’ in the United Kingdom, the largest clinical study published so far, doubts were raised about the effect of telehealthcare. Thus, the UK study concludes that telehealthcare is not a cost-effective addition to usual care [15]. However, a debate on the validity of the conclusions in the Whole System Demonstrator Project study is ongoing in Denmark for at least two reasons. First, the conclusion of the UK study might be biased since trial recruiters had foreknowledge of the allocation groups in many cases [16]. Second, its transferability to a Danish context is an issue since the trial did not consider all community and healthcare resources [15]. Addressing the latter issue is particularly important since general practitioners and municipalities are responsible for the care and monitoring of COPD patients in Denmark.

Healthcare decision makers in Denmark have therefore agreed to improve the evidence base on the effectiveness and cost-effectiveness of telehealthcare for COPD patients. As part of ‘The National Danish Action Plan for Dissemination of Telemedicine’, a full-scale, pragmatic, cluster randomized trial with a nested economic evaluation has therefore been designed for the geographical area of the North Denmark Region. This Region is one of five regions in Denmark with responsibility for healthcare for 580,000 citizens. The results of the trial will inform a political and clinical decision to implement a particular telehealthcare solution as a national standard for COPD treatment and care in Denmark [17].

The objectives of this trial are divided into two themes. The first theme has the objective to evaluate whether a particular telehealth solution that is serving as an adjunct to usual care significantly increases the health-related quality of life of patients suffering from COPD compared with usual practice. The second theme has the objective to evaluate the cost-effectiveness of this telehealth solution.

## Methods/Design

### Study design

This trial, which is known as ‘TeleCare North’ in Denmark, will be implemented in 2014and 2015. It is a full-scale, pragmatic, two-level, cluster randomized trial with 12 months of follow-up in the entire region of North Denmark Region. The municipality districts in the region define the clusters. Each municipality has between one and five districts depending on its size.

The two-level, cluster randomized, controlled study where municipality districts define the clusters was chosen for the following reasons: First, using general practitioners as both participant recruiters and randomization units could lead to selection bias concerns. To eliminate these kinds of randomization concerns, it was decided to randomize clusters at a different organizational level. Second, municipalities are organized into one or more self-contained community care districts defined by geography and personnel who provide services to COPD patients therefore usually do not overlap between districts. The cluster-randomization could therefore eliminate the risk of contamination between practices in the two alternative arms. Third, the municipality is a natural level of randomization since the responsibility for caring for and monitoring of COPD patients via the telehealth solution would eventually fall on the municipalities. Fourth, all municipalities wished to be in the telehealthcare group to provide such services at least to some of their citizens. It was therefore pragmatically decided to make these services available on a district basis within the municipalities.

### Participants

Ten out of 11 municipalities in the North Denmark Region are included in the study. One municipality (‘Læsø’) was excluded for practical reasons. The municipality has only one district and is constituted by a small island off the east coast of Jutland; it has 1,840 inhabitants (0.3% of the total inhabitants in the North Denmark Region) and one general practitioner. Matching this municipality district with other municipalities on the mainland would be difficult and questionable.

The inclusion criteria for individual participants are equivalent to those listed in the guidelines from ‘The Global Initiative for Chronic Obstructive Lung Disease (GOLD)’ [18] and other telehealthcare projects. The inclusion criteria are summarized as ‘all COPD patients that may benefit from telehealthcare’. Participants are eligible for inclusion if they have been diagnosed with COPD by spirometry and are receiving treatment as indicated in the GOLD guidelines. In addition, a general practitioner must indicate COPD as the patient’s primary disease. Patients must have a fixed residence and be listed with a general practitioner in the North Denmark Region. At least one of the following criteria should also be met: The Modified Medical Research Council scale (mMRC) ≥2 or mMRC ≥3 and COPD Assessment Test (CAT) ≥10, and have had at least two exacerbations within the past 12 months. Patients must have a phone connection and be able to speak Danish or they must be living with a Danish-speaking relative. The relative must be able to support the patient in using telehealthcare in situations where the patient cannot use the monitoring device or has problems with language or communication. Patients are excluded if they have no phone line or GSM coverage, are unable to understand Danish sufficiently to complete the study questionnaires or have a cognitive impairment.

### Recruitment

To ensure that the ten municipalities are represented in both treatment arms, the 26 municipality districts (the clusters) are paired 1:1 within the municipalities. Paired districts are paired in terms of population size, estimated proportion of citizens suffering from COPD over 40 years of age, and unemployment rate. In the municipalities of ‘Aalborg’ and ‘Frederikshavn’, the number of districts is uneven; we therefore matched the district in ‘Aalborg’ with a district in ‘Frederikshavn’ that has approximately the same population size. The COPD rate is a guess made by the trial administration office based on the number of citizens in each municipality district redeeming oral prednisolone prescriptions.

All general practitioners in these municipality districts (n = 344) are contacted by the trial administration office and invited to draw up a list of eligible COPD patients from their practice based on the inclusion criteria of the trial. At the same time, five to eight so-called ‘Telekit packages’ will be sent from the trial administration office to each practice. The ‘Telekit packages’ contain a brochure describing the trial in detail, a detailed description of patient eligibility criteria, an informed consent form for the patients to sign, a form to be filled in with baseline physical measurements, a patient-questionnaire, and two postage-paid envelopes. Voluntary information meetings targeting general practitioners are arranged at four different dates by the trial administration office. The objective, the organization of the trial, and the expectations of the participating general practitioners will be explained at these meetings.

Eligible patients are enrolled by the general practitioners. All general practitioners (supported by their local union) have agreed in advance of the trial to make individual appointments with each eligible patient listed with their practice. Before the appointment, the general practitioner posts a written folder to the patients informing them about the study and their legal rights. The appointment lasts approximately 30 minutes per patient. At the appointment, the general practitioner repeats information about the trial, hands out the same information folder that was sent to the patient before the appointment, takes baseline physical measurements (spirometry, mMRC and CAT scores, weight, pulse and blood pressure), and written informed consent is obtained if the patient accepts trial participation. If the general practitioner has obtained physical measurements less than 30 days before the appointment, these data can be used as baseline physical measurements instead.

The general practitioner sends the signed informed consent forms together with the list of eligible patients and the filled in forms with baseline physical measurements to the trial administration office using the first pre-stamped envelope. The general practitioner also distributes the patient-questionnaire which includes questions about the patients’ baseline health-related quality of life (EQ5D and SF-36) and demographic characteristics (age, gender, education, comorbidities, smoking status, marital and job status). The patient-questionnaire is filled out in the comfort of the patients’ own homes no later than two weeks after their visit to the general practitioner and must be sent to the trial administration office using the second pre-stamped envelope. The trial administration’s telephone number is printed on the questionnaires and patients are invited to contact the office if they need help to answer the questionnaire. Both envelopes are returned to the office before randomization.

Finally, the general practitioners electronically refer patients eligible for inclusion into the trial to the residing municipality where threshold values for body mass index, blood pressure, and blood oxygen saturation are established. These threshold values are individually determined for each patient and will be used in the monitoring of the patients by the municipalities’ community healthcare personnel.

### Randomization

Municipality districts are randomized so that all patients living within the same municipality district receive either telehealthcare in addition to usual care or just usual care. The randomization will not be undertaken until after all general practitioners have sent their lists of patients eligible for inclusion from their practice, and after all patients have given written consent to participation and completed baseline physical measurements and questionnaires. There will be 13 individual randomization ‘draws’ from the 26 matched municipality districts. The randomization will be done by the sealed envelope method by a person not affiliated with the trial. The randomization event will be videotaped.

### Compared alternatives

#### Usual practice

Danish healthcare is tax-funded and almost entirely publicly provided, which means that all Danish citizens and legal immigrants can access the full breadth of acute and planned medical care without any out-of-pocket payments. Five Danish Regions are responsible for supplying healthcare services to citizens, so hospitals and their staff, as a rule, are publicly owned and operated; primary care is provided via regulated contracts with independent general practitioners. The 98 municipalities in Denmark have a specialized role in rehabilitation and community care where nursing and practical help is required in the patients’ own homes.

In Denmark, usual practice for treating, monitoring, and caring for patients with COPD involves the responsibility of the patient’s general practitioner (treatment and monitoring) and that of the municipalities (practical help and nursing care). COPD patients can make appointments with their general practitioner or practice nurse free of charge in order to get help in managing their COPD. Community-based care and practical help varies across the municipalities. As a rule, community care is provided at regular intervals based on a clinical evaluation of the patients’ needs. But the personnel are not necessarily certified nurses and they are usually not particularly skilled in the handling of COPD and definitely not on a telephone call basis.

Municipality healthcare personnel in the control group will not receive the training described below, and the individual COPD patients will continue their usual treatment and care throughout the study period (12 months).

#### Intervention

The telehealth solution is a tablet (Samsung Galaxy Tab2 (10.1)) that contains information on handling COPD in general and software that automatically instructs the patient in handling COPD during exacerbations. The device can also collect and wirelessly transmit relevant disease-specific data indicative of the patient’s current state of health: blood pressure, pulse, blood oxygen saturation, and weight. This is made possible via an attached Fingertip Pulse Oximeter (Nonin, Onyx II% SpO2, A & D Medical, Tokyo, Japan), a Digital Blood Pressure Monitor (Model UA-767, plus BT-C, Nonin Medical, Minnesota, USA), and a scale (Precision Health Scale, UC-321PBT-C, A & D Medical, Tokyo, Japan). The software was developed by Silverbullit, Aarhus, Denmark (http://opentele.silverbullet.dk). The tablet can be activated and sound an alarm when it is time for taking measurements again. Patients and their relatives can access the data via a web portal (http://www.sundhed.dk).

At the cluster-level, around 100 municipality healthcare personnel (mostly community care nurses) will be responsible for patient care and monitoring in the intervention group. They will be trained by members of the trial administration office. The training consists of two full-day workshop sessions: Session 1 focuses on the technical aspects of the tablet (how to use it) and the physical measurements (how to perform the measurements using the tablet and how to monitor threshold values). Session 2 focuses on general disease awareness and communication with patients.

At the individual level, COPD patients in the intervention group will be contacted by telephone by their municipality no later than 10 days after randomization, and a 45-minute appointment is scheduled for patients who want to receive the tablet at home. For those who wish to receive the tablet at a municipality health center, a 75-minute appointment is scheduled with 3 to 4 patients in each group. A relative is allowed to participate as well. At this appointment, a nurse from the COPD patient’s municipality instructs the patient in the use of the Telekit. The nurse demonstrates how to use the tablet and how to take physical measurements, and the nurse guides the patient through the tablet manual before letting the patient try the tablet him or herself. Patients will be asked to measure their vital signs daily during the first two weeks (both weekdays and weekends) and 1 to 2 times weekly after the two first weeks (the specific days are agreed upon and programmed into the tablet by the nurse). The nurse also describes the monitoring procedure and gives the patient contact information to the community nurse. Finally, a 45-minute follow-up visit is scheduled 3 to 4 weeks after the first appointment to check if the patient uses the device appropriately and if the threshold values of the physical measurements need to be adjusted.

The patient’s residing municipality is responsible for monitoring the data obtained with the telehealth device. Exemptions are COPD patients receiving oxygen therapy and COPD patients with open hospital admissions. Such patients are monitored at a specialized hospital lung ward. Patients are monitored asynchronously by a nurse on a daily basis or no later than a weekday after each measurement. Measurements are classified with either a green, yellow or red code. A green code is given if no threshold values are exceeded. If the code given is yellow (that is, one or more values exceed the threshold values) or red (that is, one or more values exceed the threshold values and have not previously been recorded), a nurse will decide which response is necessary. The following options are available: The patient may be instructed to commence self-treatment by the tablet or the nurse can contact the patient by telephone to assess the situation, and pending the patient’s acceptance, the patient’s general practitioner can be contacted directly. Finally, an ambulance can be dispatched by the nurse.

### Outcomes

The primary outcome for theme 1 (effectiveness) is the change in health-related quality of life (SF-36) at the individual level from baseline to follow-up at 12 months. This study hypothesizes that adding telehealthcare to usual care will lead to a significantly higher health-related quality of life for the included participants.

Secondary outcomes for theme 1 are changes in mortality and physiological parameters (diastolic blood pressure, systolic blood pressure, pulse, oxygen saturation, and weight) from baseline to the follow-up at 12 months. The clinical impact of telehealthcare is assessed as being beneficial if there is no changes in mortality and if pulse and blood pressure are lowered and oxygen saturation is raised.

The primary outcome for theme 2 is the incremental cost-effectiveness ratio (ICER) measured as the cost per quality adjusted life years (QALY) gained from baseline to follow-up at 12 months. It is expected that telehealthcare will increase the patients’ QALY compared with usual practice, since no difference in mortality and a higher health-related quality of life are expected. Furthermore, it is hoped that telehealthcare will reduce the number of admissions and readmissions to hospitals as well as the number of outpatient visits by 30%, which will reduce hospital costs [13,19]. However, these savings could be offset by other costs such as more visits to general practitioners, community care, medicine consumption or the cost of implementing telehealthcare.

Outcomes will be measured at both the individual level and at cluster level.

### Sample size

The sample size calculation for theme 1 was performed in STATA v12. Based on a recent Norwegian study, it is hypothesized that the COPD patients eligible for inclusion have a mean SF-36 Physical Component Summary (PCS) score of 38 at baseline with a standard deviation of 10 [20]. The average cluster size is hypothesized to be 50 with a coefficient of variation of 0.5. Conservative estimates are applied for the minimal clinically important difference (change equal to 5) and intracluster correlation ((ICC) equal to 0.05). With a power of 80% and a two-sided *P* value of <0.05, the required sample size is estimated to be 350 participants and at least seven clusters in each arm.

With an expected loss-to-follow-up of 10%, the total required sample size is estimated to be around 800 participants.

### Statistical methods

Statistical analysis will be performed with an intention-to-treat principle and be presented according to the extensions of the CONSORT statement for cluster-randomized clinical trials [21]. Missing data (other than censoring) will be evaluated to assess the occurrence of specific missing data patterns and, if necessary, Multiple Imputation will be used and observations will be reported according to epidemiological guidelines [22]. If appropriate, an adjustment for differences in patient and/or cluster baseline characteristics (for example, age, gender, co-morbidities, educational level, marital status, proportion of COPD patients) in both the telehealthcare group and in the usual care group will be made.

To assess effectiveness in theme 1, a comparison of differences in mean SF-36 scores, mortality and physical measurements from baseline to 12 months after randomization between the telehealthcare group and the control group will be analyzed using statistical methods suitable for cluster-randomized trials, for example, multilevel modeling.

To assess the cost-effectiveness in theme 2, a point estimate of the ICER will be calculated after 12 months. The ICER is defined as the difference in arithmetic mean costs between the telehealthcare intervention and usual care divided by the difference in arithmetic mean effectiveness between the same intervention groups as shown in Equation (1).

Equation 1 shows how the incremental cost-effectiveness ratio (ICER) is calculated:

(1) $$\mathrm{ICER}=\frac{C_{\text{Telehealthcare}}-C_{\text{Usual}\;\text{care}}}{E_{\text{Telehealthcare}}-E_{\mathrm{Usual}\;\text{care}}}$$

The incremental effectiveness for the cost-effectiveness calculation is estimated from the utility scores from the EQ5D-3 L health-related quality of life questionnaire with Danish societal weights [23]. From these, QALYs can be calculated by linear interpolation of utility scores between baseline and the follow-up at 12 months by measuring the area under this curve.

In calculating the incremental costs, this trial adopts a healthcare sector perspective in the base case analysis, which means that all costs to healthcare providers involved in treating, caring, and monitoring COPD patients are included [24]. Included cost categories are intervention costs, in- and outpatient costs, medication costs, costs from visiting general practitioners, and community care costs (primarily due to home nurse visits in the municipalities). This choice of perspective for the base case analysis means that the calculation of costs per QALY excludes costs incurred by patients and relatives (primarily due to transportation and time) and costs due to productivity loss to society. Excluding cost of productivity loss is normal practice in economic evaluation when conducting cost-utility analysis because of the risk of double counting [24]. To allow for later sensitivity analysis, information on productivity loss is gathered at 12 months by use of the Work Productivity and Activity Impairment questionnaire (WPAI) [25]. The WPAI measures self-reported absence from work, reduction in productivity while at work, and reduction in productivity while doing regular activities (other than work) in order to be able to assess the cost-of-illness to society at follow-up. Costs to patients and relatives are sought estimated from the literature.

Denmark is internationally recognized for having a rigorous registration of data on health activities. All Danish citizens have a social security number, which is a 10-digit unique identity number. All Danish citizens’ contacts with the healthcare sector are registered in various registers with this identification number. Through cross-linking of registers, data may be extracted at the level of the individual to obtain a fairly accurate picture of the individual's consumption of health care resources/services across municipalities, hospitals, and general practitioners.

Three national registers are relevant in the present study: Firstly, *The Danish National Patient Register* contains national patient-level data for all hospital contacts (patient cpr-number, date of beginning/end of contact, diagnoses, treatments, and place of contacts). The register contains data on all inpatient, outpatient, and emergency ward visits in Denmark. Secondly, *The National Health Insurance Service Register* covers all contacts between patients and the primary care sector (general practitioners, physiotherapists, *etcetera*). The health authorities in Denmark use the register for administrative purposes, especially for the settling of accounts. The register contains data on all citizens, providers, and healthcare services reimbursed by the health authorities. Thirdly, *The Register of Medicinal Product Statistics* contains detailed information about the total sales of prescribed medicine in Denmark. Around 30 different items of information are registered every time a medicinal product is sold on prescription.

Finally, data on the use of community care is collected from the individual care systems in the residing municipalities and implementation cost are collected from cost diaries from the trial administration office.

Unit costs will be calculated for each of the resource parameters in order to determine the average costs per patient at baseline and at the 12-month follow-up. Point estimate for the average costs in the telehealthcare group and in the usual care group are then derived from univariate and multivariate analysis.

The cost-effectiveness analysis will be subject to both one-way and probabilistic sensitivity analysis (PSA). This is a standard way of analyzing and presenting results from a cost-effectiveness calculation that allows the recipients of the analysis to get a sense of the impact of different uncertainties in parameter values on the probability that telehealthcare is cost-effective at different levels of willingness to pay for an improvement in QALYs [26,27]. Another trial ambition is to expand the healthcare sector view to include costs incurred by patients and relatives (primarily due to transportation and time) and costs due to productivity loss to society that were excluded in in the base case scenario of total cost per QALY.

### Ethics

The study is conducted in accordance with the Helsinki Declaration. The trial has been presented to the Regional Ethical Committee for Medical Research in the North Denmark Region where it was determined that no ethical approval was necessary. The trial has also been authorized by the Danish Data Protection Agency.

## Discussion

There has been a call for large-scale clinical trials with rigorous cost-effectiveness assessments in telehealthcare research. This study is meant to improve the international evidence base for the effectiveness and cost-effectiveness of telehealthcare to patients suffering from COPD by implementing a large-scale pragmatic cluster-randomized clinical trial throughout the geographical area of North Denmark Region. Changes in health-related quality of life and costs are evaluated using a broader range of cost categories than most existing studies.

For practical and ethical reasons, the involved stakeholders decided that all eligible patients and municipality districts in North Denmark Region should be able to option for the telehealthcare intervention no later than 12 months after randomization. It could be argued that the 12 month timeframe of the trial may be too short to capture all future costs and outcomes since COPD is a chronic disease. On the other hand, there is to our knowledge no evidence to suggest an appropriate timeframe for including all relevant future costs and clinical outcomes. However, a modelling study extrapolating QALY differences over a longer period could be considered to supplement the economic evaluation performed in this clinical trial.

The expectation is that the results of the trial can be generalized to all municipalities in Denmark. ‘The National Danish Action Plan for Dissemination of Telemedicine’ has determined that if the telehealthcare solution in this trial proves cost-effective, it can serve as a national Danish standard for a technological platform as well as an implementation model for telehealthcare to this patient group. The technical platform is therefore not patented, and codes and documentation of how the study was implemented will be published.

## Trial status

Recruitment is ongoing.

## Abbreviations

COPD: chronic obstructive pulmonary disease; ICER: incremental cost-effectiveness ratio; QALY: quality adjusted life years

## Competing interests

The authors declare that they have no competing interests.

## Authors’ contributions

LHE contributed to the conception of the study, the overall design and fundraising, critical revision and final approval of the manuscript. OH contributed to the conception of the study, the overall design and fundraising, critical revision and final approval of the manuscript. PHL contributed to the detailed design of the study, manuscript writing (in particularly the paragraphs describing the intervention and effectiveness outcomes and -analysis), critical revision and final approval of the manuscript. FWU contributed to the detailed design of the study, all manuscript writing, critical revision and final approval of the manuscript. All authors read and approved the final manuscript.

## Authors’ information

FWU is a PhD student at the Danish Center for Healthcare Improvements at Aalborg University. PHL is a PhD student at the Department of Health Science and Technology, Aalborg University. OH is a professor at the Department of Health Science and Technology, Aalborg University. LHE is a professor in health economics at the Danish Center for Healthcare Improvements at Aalborg University.
